# Enhanced Corrosion Performance of Epoxy Coatings Painted on ZnAlMg-LDH Conversion Film Vertically Grown on ZAM Steels from Sodium Carbonate Solution

**DOI:** 10.3390/molecules30173491

**Published:** 2025-08-25

**Authors:** Lei Yu, Ji-Ming Hu

**Affiliations:** Department of Chemistry, Zhejiang University, Hangzhou 310058, China; 22337074@zju.edu.cn

**Keywords:** zinc-aluminum-magnesium (ZAM) steel, layered double hydroxide (LDH), vertically grown, pretreatment layer, sodium carbonate solution, adhesion, corrosion performance

## Abstract

Zinc-aluminum-magnesium (ZAM) steel, with its superior corrosion resistance and mechanical properties, is progressively supplanting traditional galvanized steel and zinc-aluminum steel. In this study, a solution containing sodium carbonate-only was employed as the treatment medium to form a vertically grown layered double hydroxide (LDH) pretreatment layer on the surface of ZAM steel via a simple immersion process at 50 °C. The temperature and salt solution not only provide the conditions for the dissolution of metal ions but also facilitate the formation of LDH products. The resulting LDH pretreatment layer exhibits excellent adhesion to the metal surface and enhances the adhesion of the top epoxy coatings. Furthermore, the “LDH/corrosion inhibitor/epoxy” coating system ensures ZAM steel remains rust-free in a 3.5 wt.% NaCl solution for a minimum of 120 days. This innovative approach offers a promising avenue for extending the durability and service life of ZAM steel in corrosive environments.

## 1. Introduction

Metallic materials, serving as widely utilized structural materials in human society, have a profound impact on public safety and social development through their susceptibility to corrosion. According to statistics, corrosion causes losses of at least 2.3 trillion US dollars annually [1,2]. As the most commonly employed method for metal protection, coating systems play a critical role in mitigating corrosion [3,4]. The pretreatment layer, together with organic resins, form the protective coating system [5,6]. Acting as the innermost protective structure adjacent to the substrate, the pretreatment layer not only provides highly effective active protection [7,8,9] but also enhances the adhesion between the topcoats and the metal substrate, thereby imparting greater durability and persistent barrier properties to the topcoat. Traditional pretreatment layers include phosphate coatings and chromate conversion coatings. These treatments offer commendable corrosion resistance to the substrate. However, the use of chromate conversion coatings releases hexavalent chromium ions, which are highly hazardous to human health when they enter the ecosystem. Meanwhile, phosphates used in phosphate coatings contribute to eutrophication in water bodies [10,11,12,13], thus limiting their practical applications. In light of these drawbacks, there is an urgent need within both the scientific community and industry for an environmentally friendly and high-performance pretreatment layer that can address these issues.

Layered double hydroxides (LDHs) have emerged as a novel class of layered materials in recent years. Their general chemical formula can be expressed as [M^2+^_1−x_M^3+^_x_(OH)_2_]^x+^A^n−^_x/n_·mH_2_O, where M^2+^ and M^3+^ denote divalent and trivalent metal cations, respectively, which occupy the octahedral interstices of brucite-like hydroxide layers, thereby forming the metal hydroxide layer [14]. A^n−^ represents the anions intercalated within the LDH interlayers. These interlayer anions exhibit a certain degree of exchangeability, making LDHs suitable as nanocontainers for encapsulating corrosion inhibitors [15,16]. Additionally, the two-dimensional planar nature of LDHs confers them with effective ion-blocking capabilities against corrosive species [17,18]. As a rising star in the field of corrosion protection, LDHs find application primarily in two aspects: one is that, they can be incorporated directly into organic resins as “nano-containers” to serve as fillers [17,19]; the other is that, LDHs can be deposited on the substrate surface, functioning either as short-term protective films for metals [20,21] or as pretreatment layers for topcoats [22,23].

For the purpose of their use as a pretreatment layer, LDH films better exhibit a morphology that the (*ab*) plane can vertically to the metal substrate, so that the conversion coatings can provide improved adhesion to the top-coated coatings. The conventionally used hydrothermal method satisfies such requirements [24,25], however, it needs elevated temperatures and has prolonged reaction times [26,27,28]. This is detrimental to reactive elemental metals, as the metal substrate would undergo severe corrosion under such circumstances. The electrochemical deposition method needs a lower temperature and shorter preparation time, the as-prepared LDH coating is always flat and compact with the (*ab*) plane parallel to the substrate. Therefore, the electrodeposited LDH is hardly used as a pretreatment layer for organic coatings. Very recently, our laboratory [29] utilized a multi-potential-step electrodeposition approach to prepare a LDH pretreatment layer on galvanized steel, via the in situ generation of Zn^2+^ ions by anodic dissolution in the first step, and the formation of ZnAl-LDH with the participation of Al^3+^ ions that already existed in the solution by applying cathodic potentials in the second step. LDH coatings prepared by this two-step electrodeposition have a morphology with the (*ab*) plane perpendicular to the galvanized steel surface, but this technique needs additional energy input and equipment; therefore, proposing a mild, convenient, and rapid preparation method for the LDH pretreatment layer becomes particularly significant.

ZnAlMg (ZAM) coated steel combines the high strength of structural steel with the robust anti-corrosion performance provided by the coating, leading to its extensive use in automotive bodies and bridge frameworks, among other structures [30,31]. In spite of this, ZAM steels still require further improvement to their corrosion resistance by, for instance, applying polymeric coatings; this is urgent in harsh corrosive conditions, such as in marine environments. Here, a simple, efficient, and rapid method is introduced for the one-step preparation of an LDH pretreatment layer on ZAM steel using a precursor solution with the absence of any exogenous LDH-forming metal ions. The hot sodium carbonate solution not only provides a dissolution environment for Zn, Al, and Mg metals but also facilitates the formation of LDHs. Given that all metal ions originate from the ZAM steel itself, the in situ grown LDH coatings ensure excellent adhesion to the metal substrate. Moreover, the vertically oriented morphology (with ab-faces perpendicular to the substrates) of the as-prepared LDH conversion coatings ensures strong adhesion with the subsequent polymeric coatings and enables the pre-incorporation of the desired amount of corrosion inhibitors beneath the topcoat. The results demonstrate that the “LDH/corrosion inhibitor/epoxy” coating system prevents rust formation on ZAM steel in a 3.5 wt.% NaCl solution for at least 120 days. The synthesis of LDH requires only three min and does not necessitate the addition of any external film-forming metal ions.

## 2. Results

### 2.1. Characterization of LDH Pretreatment Layer

**Figure 1:**
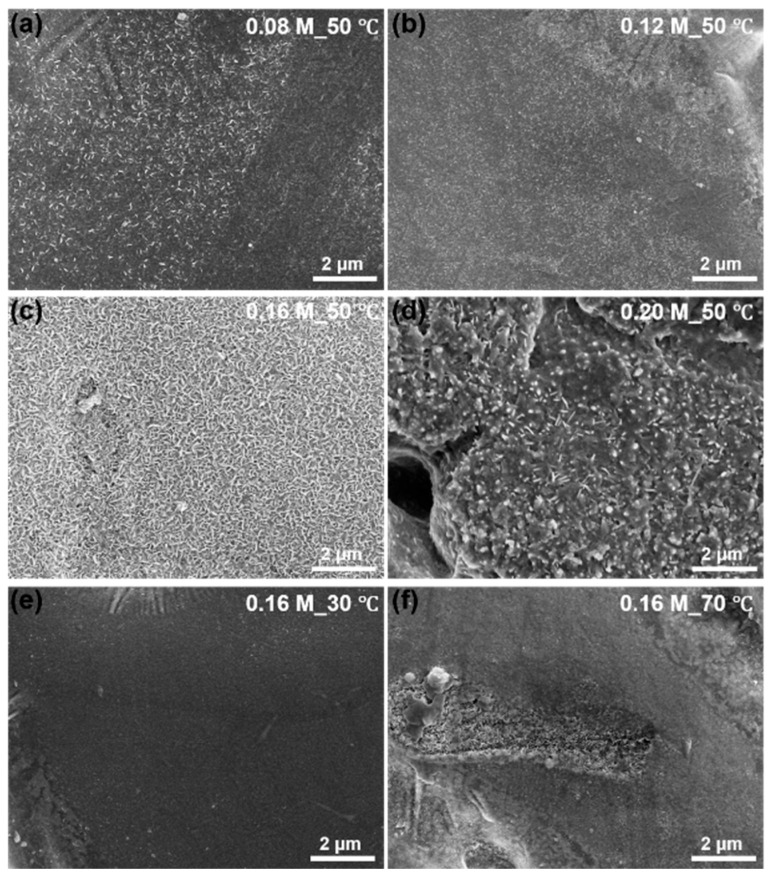
SEM images of LDH conversion coatings prepared at a constant temperature of 50 °C with different Na_2_CO_3_ solution concentrations: 0.08 mol/L (**a**), 0.12 mol/L (**b**), 0.16 mol/L (**c**), and 0.20 mol/L (**d**); and at a constant concentration of 0.16 mol/L with different temperatures: 30 °C (**e**) and 70 °C (**f**).

The surface morphology of ZAM steels after treatment in Na_2_CO_3_ solutions of various concentrations at 50 °C (a–d) and at a concentration of 0.16 M at different temperatures (e,f) is illustrated in Figure 1. At lower concentrations and temperatures, LDH formation is either partial (a,b) or does not occur on the ZAM substrate (e). Conversely, when the concentration or temperature of the Na_2_CO_3_ solution is high enough, the LDH exhibits dissolution behavior (d,f). The dissolved LDH transforms into oxides or hydroxides that cover the original LDH coatings. Optimal conditions, like 0.16 M Na_2_CO_3_ + 50 °C, lead to a uniform and high-coverage LDH coating. Unless otherwise mentioned, LDH conversion coatings in this work were prepared from 0.16 M Na_2_CO_3_ at 50 °C.

**Figure 2:**
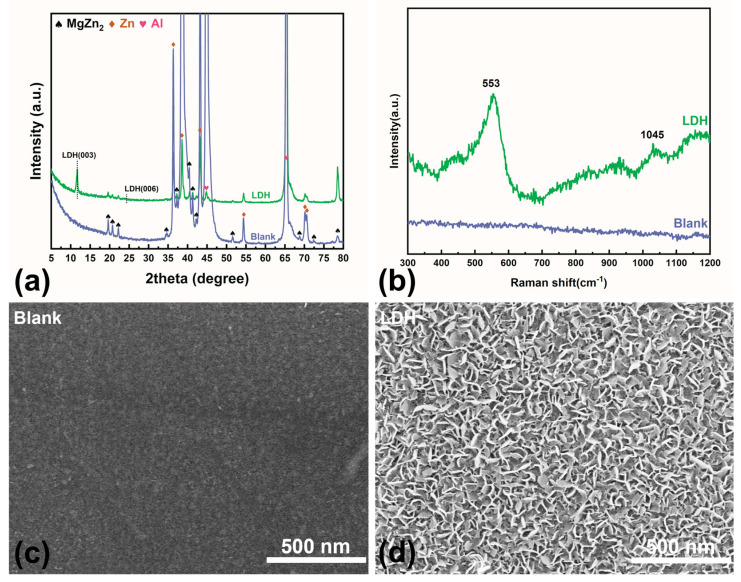
(**a**) XRD patterns of bare ZAM steel and LDH-coated ZAM steel; (**b**) Raman spectra of bare ZAM steel and LDH-coated ZAM steel; (**c**,**d**) SEM images of LDH-coated ZAM steel and bare ZAM steel.

The XRD patterns of bare ZAM steel and LDH-coated ZAM steel are presented in Figure 2a. In the untreated ZAM steel, no distinct diffraction peaks characteristic of LDHs, specifically the (003) and (006) peaks, were detected. The XRD pattern of this blank sample primarily consists of Zn peaks at 36.27°, 43.27°, and 77.46°, and Al peaks at 38.39°, 44.68°, 65.08°, and 77.88°. Additionally, there are some characteristic peaks of the intermetallic phase MgZn_2_ [32]. After treatment using the aforementioned method, characteristic LDH peaks appeared on the ZAM steel surface at 11.66° (003) and 24.22° (006) [33], indicating the formation of a well-crystallized LDH film. The Raman spectra align well with the XRD results (Figure 2b). For the bare ZAM steel, no significant signals were observed, resulting in a nearly flat baseline. After treatment, two prominent peaks attributed to LDH appear on the spectrum at 553 cm^−1^ and 1045 cm^−1^ [34].

SEM images show that, compared to the blank sample (Figure 2c), the LDH sample (Figure 2d) exhibits a finely textured rough structure. The lamellar LDH grows vertically on the metal surface, endowing the ZAM steel with nanoscale pores and grooves. These nanostructures enable the pretreatment layer to absorb numerous corrosion inhibitors that are beneficial for enhancing the corrosion resistance of the metal. Additionally, they can interlock with subsequent polymeric resins, facilitating coating adhesion [9,29].

**Figure 3:**
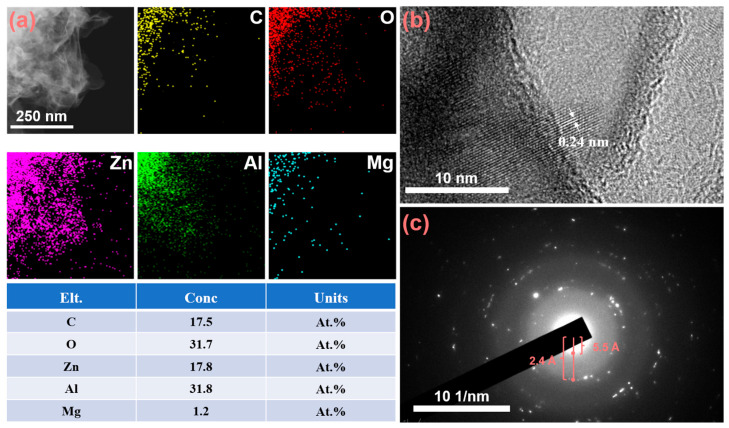
(**a**) EDS mapping results of the LDH coating grown on ZAM steel, (**b**) High-resolution TEM (HRTEM) image (**c**) Electron diffraction pattern of LDH coating.

The samples were subjected to ultrasonic treatment, and the resulting LDH was examined using transmission electron microscopy (TEM). As shown in Figure 3a, Energy-Dispersive X-ray Spectroscopy (EDS) mapping results indicate that the LDH primarily consists of Zn, Al, C, and O, with trace amounts of Mg. Among these elements, C and O predominantly originate from the Na_2_CO_3_ solution used during preparation, which provides CO_3_^2−^ ions as intercalation ions for LDH formation. During the treatment of ZAM steel with a hot sodium carbonate solution, Zn, Al, and Mg undergo in situ transformation into hydroxides and soluble species such as Zn(OH)_4_^2−^ and Al(OH)_4_^−^. In the formation process of LDH, trivalent metal ions Al^3+^ partially substitute divalent metal ions Zn^2+^ within the layers, imparting an excess positive charge to the layer sheets. The carbonate ions enter the interlayer region via electrostatic interactions, neutralizing these positive charges and maintaining the overall charge neutrality of the LDH structure. A high-resolution TEM (HRTEM) image (Figure 3b) clearly displays the lattice fringes of the LDH. The inter-planar spacing of 0.24 nm corresponds to the (012) crystal plane of LDH. As shown in Figure 3c, the selected-area electron diffraction (SAED) pattern reveals the diffraction rings, indicating that the formed LDH possesses a highly ordered crystalline structure. These distinct diffraction rings correspond to different lattice planes within the crystal structure. Notably, the broadening at 2.4 Å and 5.5 Å corresponds to the (012) and (003) planes of the LDH. Figure S1 presents HRTEM images and EDS point scan results of LDH at lower magnification. From Figure S1a, it is evident that LDH exhibits a pronounced lamellar structure. The EDS analysis in Figure S1b indicates that the Zn/Al ratio of the formed LDH is approximately 2:1, which is the most favorable metal ratio for LDH formation on ZAM steel during natural corrosion [35].

**Figure 4:**
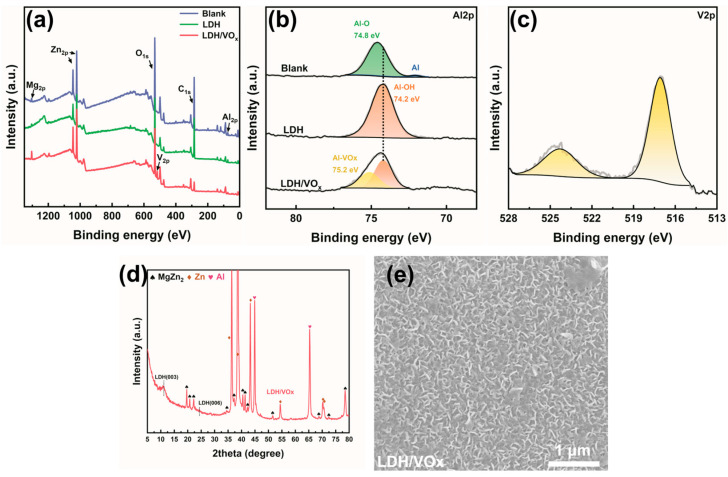
(**a**) Full scan and (**b**) Al 2p XPS spectrum of bare ZAM steel, LDH-coated ZAM steel, and LDH-coated ZAM steel after the adsorption of corrosion inhibitors, (**c**) V 2p XPS spectrum, (**d**) XRD patterns, (**e**) SEM image. The corresponding phases in the XRD pattern have been labeled in the upper left corner.

XPS spectra (Figure 4a) shows the presence of Zn, Al, Mg, C, and O elements in blank and LDH-coated ZAM coatings. In the results of the inhibitor-loaded samples, a distinct V signal is also observed. Figure 4b presents the Al 2p spectrum, which distinctly reflects the chemical state of Al on the substrate surface. For the blank sample, the Al 2p peak can be fitted into two components: one at approximately 72.3 eV corresponding to the Al-Al bond in metallic Al on the bare ZAM steel surface, and another around 74.8 eV attributed to the Al-O bond in Al_2_O_3_ [36]. Due to its high reactivity, an aluminum layer or alloy exposed to air typically develops a dense oxide layer that prevents further oxidation of the underlying metal. After treatment with a hot Na_2_CO_3_ solution (LDH sample), a new peak appears at 74.2 eV for Al-OH, completely replacing the 74.8 eV Al-O peak, indicating complete coverage of the ZAM surface by hydroxides. For the inhibitor-loaded sample (LDH/VOx sample), an additional peak at a higher binding energy of 75.2 eV is observed in the Al 2p spectrum, alongside the 74.2 eV Al-OH peak. This higher binding energy peak arises from the introduction of highly electronegative VO_3_^−^ ions, which form complexes with metal ions, resulting in a blue shift of the binding energy. Figure 4c shows the V 2p peaks characteristic of the LDH/VOx sample, with two peaks at 524.3 eV for V 2p1/2 and 517.1 eV for V 2p3/2, confirming the successful loading of the VO_3_^−^ inhibitor onto the LDH pretreatment layer. The XRD pattern of the LDH/VOx sample (Figure 4d) detected a broader (003) peak of LDH, compared with the LDH sample, indicating a decrease in crystallinity after inhibitor loading. The SEM image in Figure 4e corroborates this observation, showing a certain degree of collapse in the morphology of the LDH/VOx sample.

### 2.2. Corrosion Performance of the LDH Pretreatment Layer and the Whole Coating System

**Figure 5:**
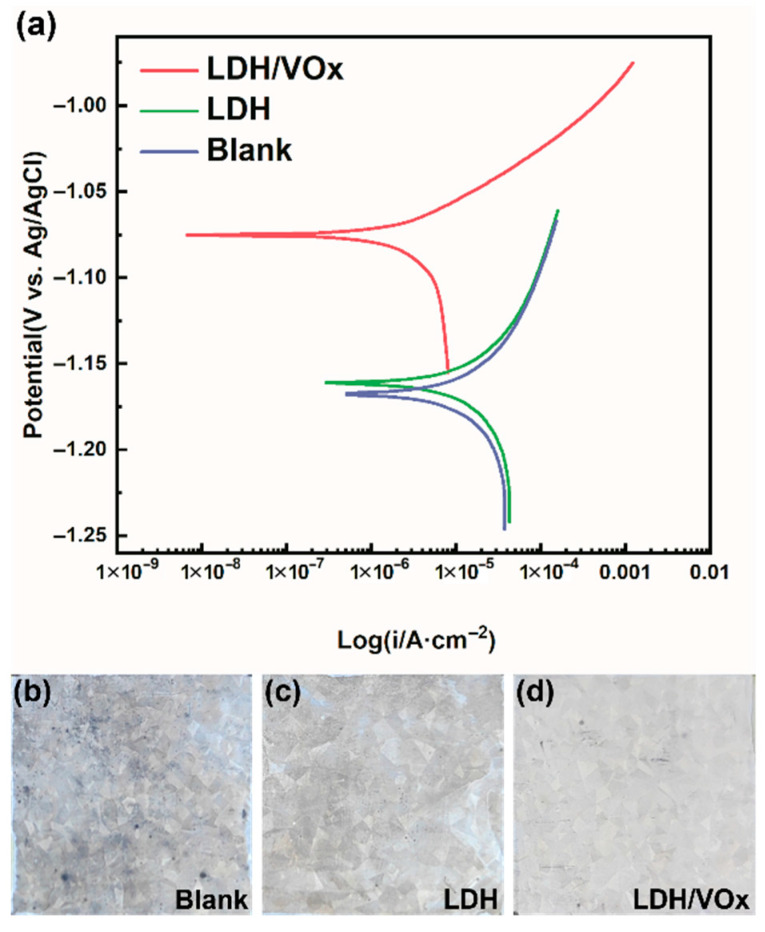
(**a**) Potentiodynamic polarization curves of different samples after immersed in a 3.5 wt.% NaCl aqueous solution for 30 min; the surface morphology of (**b**) blank, (**c**) LDH, and (**d**) LDH/VOx sample after immersion in a 3.5 wt.% NaCl solution for 24 h.

The potentiodynamic polarization curves (Figure 5) show that, the blank ZAM steel exhibits the highest corrosion current density (Icorr, 1.60 × 10^−5^ A/cm^−2^, Table 1) among all samples, along with the most severe corrosion morphology (Figure 5b). After coated with a LDH conversion layer, Icorr slightly decreases to a value of 1.21 × 10^−5^ A/cm^−2^ (Table 1). The decrease is negligible, probably because of the vertical orientation of the LDH lamella; however, the corrosion morphology of the LDH sample showed significant improvement (Figure 5c). The lowest Icorr is observed in the LDH/VOx sample, which is approximately one order of magnitude lower than that of both the blank and LDH samples. The loading of the corrosion inhibitor VO_3_^−^ significantly enhances the corrosion inhibition performance of the LDH coating, markedly improving the corrosion resistance of ZAM steel in a 3.5 wt.% NaCl aqueous solution (Figure 5d). It is quite evident that the corrosion rate ranking of the three samples is: Blank > LDH > LDH/VOx.

**Figure 6:**
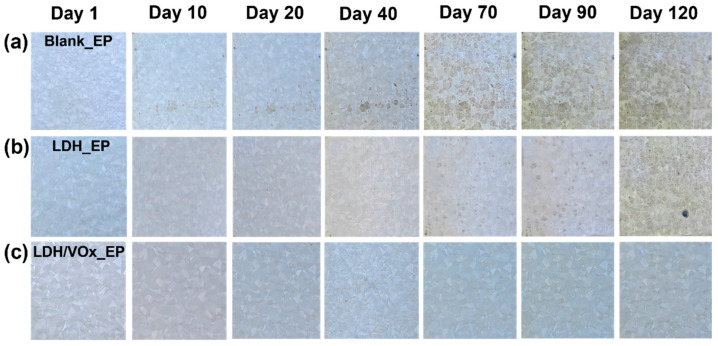
Optical images of the intact coating samples immersed in a 3.5 wt.% NaCl solution for 120 days. (**a**) Blank_EP, (**b**) LDH_EP, (**c**) LDH/VOx_EP.

Long-term immersion tests in a 3.5 wt.% NaCl solution were used to evaluate corrosion performance of epoxy coating samples (Figure 6). Noticeable rust appears beneath the coatings on blank ZAM substrates (Figure 6a). As the immersion time extended, these initial corrosion spots progressively expanded and covered the entire surface after 70 days. These results indicate that the pure epoxy coating alone cannot provide ZAM steel with durable and effective protection. In contrast, the LDH_EP sample (Figure 6b) did not show any corrosion phenomena until day 70, and even then, the extent of corrosion was significantly less severe compared to the Blank_EP sample. This suggests that the presence of the LDH pretreatment layer enhances the corrosion resistance of epoxy-coated ZAM steel. The superior corrosion resistance performance was most evident in the LDH/VOx_EP sample. Even after 120 days of immersion, its surface remained free from any visible corrosion. The LDH coatings loaded with VO_3_^−^ further augmented the anti-corrosion performance of the steel. The long-term immersion test results demonstrate that while the pure epoxy coating provides limited protection, the incorporation of an LDH pretreatment layer markedly improves the corrosion resistance of ZAM steel. Moreover, the addition of the VO_3_^−^ inhibitor within the LDH layer offers significant enhancement in protecting the steel substrate against corrosion, highlighting the effectiveness of this composite coating system.

**Figure 7:**
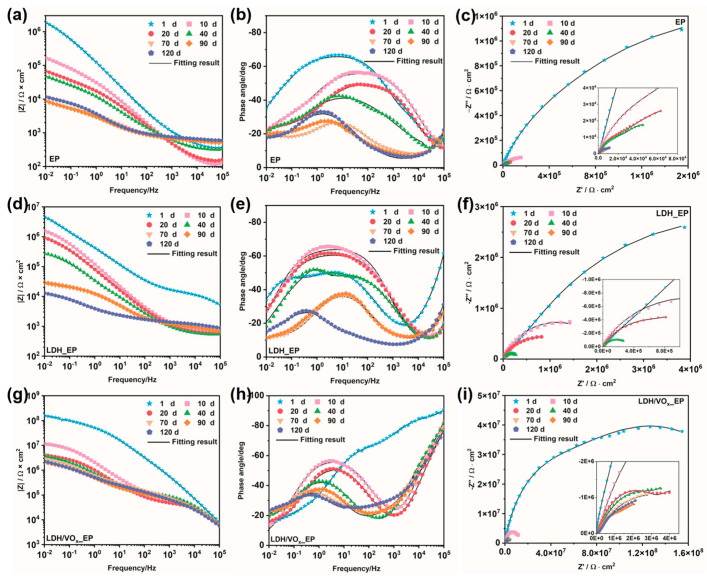
Impedance module-Bode plots (**left**), phase angle-Bode plots (**middle**), and Nyquist diagram (**right**) of the intact coating samples immersed in a 3.5 wt.% NaCl solution. (**a**–**c**) EP; (**d**–**f**) LDH_EP; (**g**–**i**) LDH/VOx_EP.

**Figure 8:**
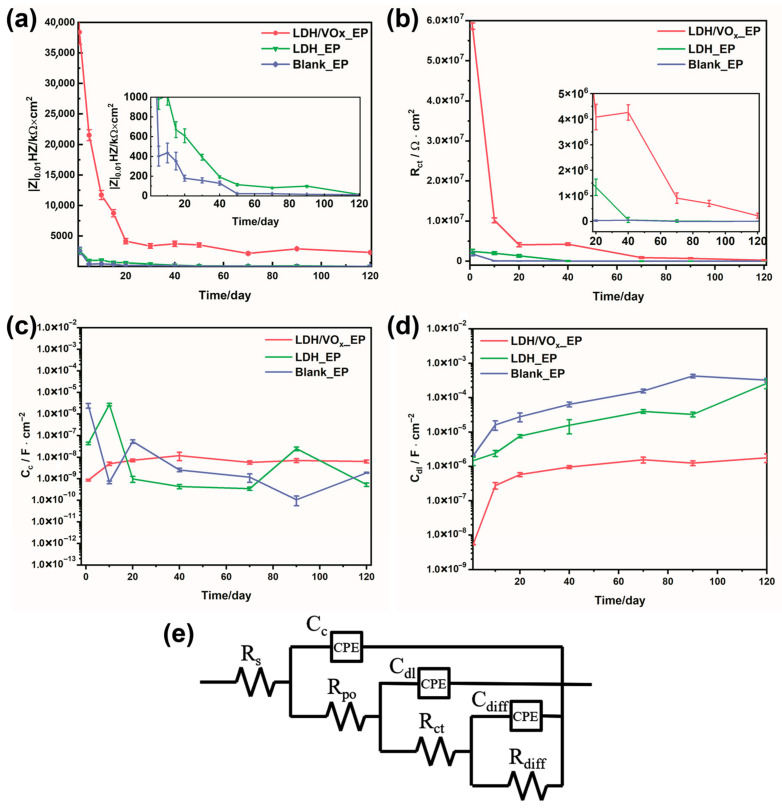
(**a**) The variation in the low-frequency impedance modulus of the intact coating in a 3.5 wt.% NaCl solution; the variation in (**b**) R_ct_, (**c**) C_c_, (**d**) C_dl_ over the entire immersion period; (**e**) the Equivalent electrical circuit model (EEC) used for fitting EIS data.

EIS experiments were employed to quantitatively evaluate the corrosion resistance of the coating systems and to further analyze the corrosion mechanisms of the coatings. The impedance modulus-Bode plots (left), phase angle-Bode plots (middle), and Nyquist diagrams (right) for intact coated samples immersed in a 3.5 wt.% NaCl solution are presented in Figure 7. The low-frequency impedance values of all samples (Figure 8a) continuously decreased throughout the immersion period, indicating a gradual deterioration of the barrier properties of the epoxy coatings. For the EP sample, even at the initial stages of immersion (Figure 7a), the impedance value was approximately 10^6^ Ω·cm^2^; by the end of the immersion period (90–120 days), this value had declined to around 10^3^ Ω·cm^2^. The relatively low initial impedance and the rapid decline suggest that, without the synergistic effect of an LDH pretreatment layer, the protection provided by epoxy resin to ZAM steel is quite limited. In contrast, the LDH_EP sample exhibited higher impedance values (Figure 7d) and larger semicircle diameters (Figure 7f) compared to the EP sample at the same immersion times, indicating that the formed LDH pretreatment layer can enhance the anti-corrosion performance of subsequent coatings to some extent. Consistent with previous results, the coating system prepared after loading the LDH with a corrosion inhibitor demonstrated the best anti-corrosion performance. The largest semicircle diameter (Figure 7i) and highest impedance values (Figure 7g) were observed for the LDH/VOx_EP samples. Throughout the entire immersion period, the impedance values remained above 10^6^ Ω·cm^2^, providing sustained excellent protection for the ZAM steel substrate.

An appropriate Equivalent Electrical Circuit (EEC) was selected to fit the EIS data (Figure 8e). This EEC, which contains three time constants, was applicable for fitting throughout the entire immersion period. For the first time constant, R_po_ and C_c_ represent the pore resistance and coating capacitance, respectively, after the electrolyte solution has penetrated the coating [37]. When the electrolyte reaches the substrate surface, electrochemical reactions occur at the metal/coating interface, introducing the second time constant. R_ct_ and C_dl_ denote the charge transfer resistance and double-layer capacitance at the electrochemical reaction interface, respectively [38]. The third time constant is associated with the products of the electrochemical reactions. Its presence is linked to the accumulation of reaction products at the metal/coating interface. R_diff_ and C_diff_ are the diffusion resistance and diffusion capacitance of the coating [29]. It is noteworthy that the EEC employing R_diff_ and C_diff_, characterized by Finite-Length Diffusion (FLD), has been widely adopted for fitting impedance data of coated samples after enhanced immersion [39]. All capacitive elements in the circuit were replaced with Constant Phase Elements (CPEs) to achieve accurate fitting results. Equation (1) defines the impedance of a CPE, where *Y_0_* is the magnitude of the CPE, ω is the angular frequency, and *n* is a dimensionless parameter.

(1) $$Z_{CPE}=\frac{Y_{0}^{-1}}{{\left(j\omega \right)}^{n}}$$

The values of R_ct_ and C_dl_ are closely related to the degree to which metal corrosion is inhibited. Lower C_dl_ and higher R_ct_ indicate better protection of the metal in the corrosive medium. Figure 8b,d shows that the LDH/VOx_EP sample maintained the highest R_ct_ values and the lowest C_dl_ values throughout the entire immersion period, indicating superior protective performance provided by the inhibitor-loaded pretreatment layer. In contrast, the EP sample exhibited the highest C_dl_ values and the lowest R_ct_ values. The continuously increasing trend of C_dl_ suggests a growing interface between the coating and the metal during immersion. This expansion is attributed to rapid corrosion, leading to the accumulation of significant amounts of corrosion products at the interface, which promotes delamination between the metal and the coating. The LDH_EP sample showed intermediate behavior between the two extremes, indicating that the LDH pretreatment layer can enhance the corrosion resistance of the coating system to some extent. The water absorption of the coating can typically be assessed by the coating capacitance C_c_, lower values indicate greater diffusion resistance to water and, consequently, lower water absorption. Figure 8c presents the C_c_ values for all samples. During the 120-day immersion test, all samples exhibited similar C_c_ values. This similarity in C_c_ suggests that, despite differences in their protective performance against corrosion, the coatings have comparable resistance to water diffusion. However, it is important to note that while C_c_ provides insight into water absorption, it does not fully capture the complex interactions between the coating and the corrosive environment, which are also influenced by factors such as the presence of protective layers and inhibitors. Overall, the corrosion rate ranking of the coated samples is as follows: Blank_EP > LDH_EP > LDH/VOx_EP.

**Figure 9:**
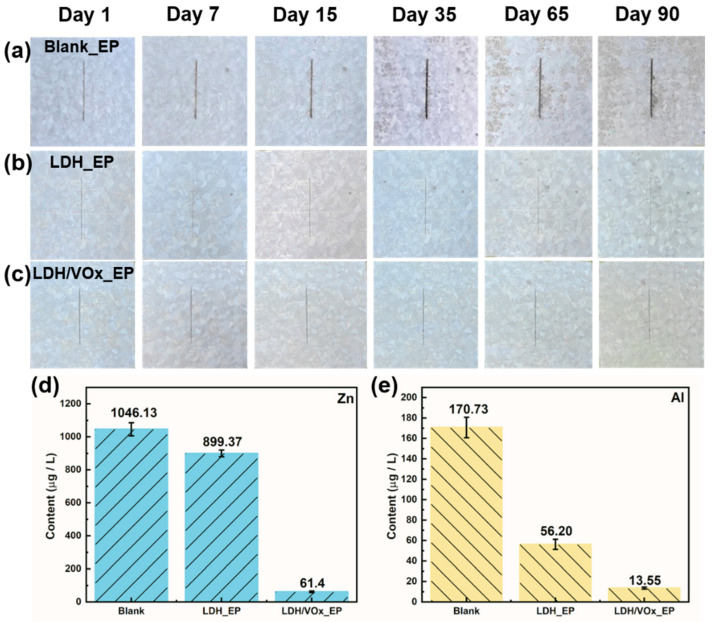
Optical images of the scratched Blank_EP sample (**a**), LDH_EP sample (**b**), and LDH/VOx_EP sample (**c**) immersed in a 3.5 wt.% NaCl solution for 90 days. (**d**) Zn and (**e**) Al content in the solution after 90 days of immersion of scratched coating samples.

Artificial defects were introduced on intact coated samples, which were then immersed in a 3.5 wt.% NaCl solution. Optical photographs of all samples at various immersion durations are shown in Figure 9a–c. On the first day of immersion, rust began to appear along the scratch on the blank sample (Figure 9a). As the immersion time extended, corrosion spots appeared by day seven, with extensive corrosion marks evident by day thirty-five. By the end of the immersion period, corrosion products had accumulated extensively at the metal/coating interface, indicating that the epoxy coating (EP) offered limited protection to ZAM steel. For the LDH_EP sample (Figure 9b), corrosion spots also appeared by day seven but did not expand further with prolonged immersion. Even after 90 days, only sporadic corrosion products were observed. After mechanical damage to the coating, the LDH pretreatment layer effectively inhibited the corrosive medium from attacking the metal/coating interface. The lamellar structure of LDHs significantly increased the lateral diffusion resistance of the medium [40]. When loaded with a corrosion inhibitor, the protective performance of the pretreatment layer was further enhanced. The synergistic effect of active protection (inhibitor action) and suppression of lateral electrolyte diffusion resulted in the LDH/VOx_EP sample showing corrosion traces only by day 65 (Figure 9c). Subsequent immersion did not lead to an expansion of corrosion spots or additional corrosion. The coating system composed of LDH/VOx and subsequent epoxy resin significantly improved the corrosion resistance of ZAM steel. Figure 9d,e shows the amounts of Zn and Al ions leached due to corrosion after 90 days of immersion. The results indicate that the LDH/VOx_EP sample has the lowest levels of Zn and Al ion release, followed by the LDH_EP sample. The presence of the LDH pretreatment layer greatly restricted the lateral spread of the electrolyte solution, preventing corrosion products from spreading outward from the defect site even when the EP coating was damaged. In contrast, the EP sample exhibited the highest ion release, consistent with the previously summarized corrosion resistance ranking.

**Figure 10:**
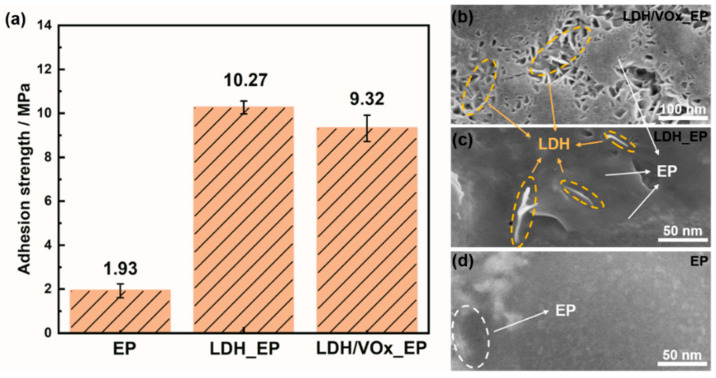
(**a**) Pull-off test results of different samples; (**b**–**d**) SEM images of different samples after pull-off test.

The pull-off test results for all samples are presented in Figure 10a. On the bare ZAM steel substrate, the adhesion strength of the epoxy (EP) coating was only 1.93 MPa. When a pretreatment layer was incorporated into the coating system, the adhesion strength of the EP significantly increased to 10.27 MPa for the LDH_EP sample and 9.32 MPa for the LDH/VOx_EP sample. Figure 10b–d show the SEM images of different samples after the pull-off tests. On substrates with LDH pretreatment layers, the lamellar LDH structures interlocked with the EP coating, forming a “concave-filled structure” that substantially enhanced the adhesion strength of the EP. In contrast, the EP sample exhibited extensive delamination due to its lower adhesion strength. The interlocking mechanism between the LDH pretreatment layer and the EP coating is evident from the SEM images, where the lamellar LDHs are observed to be tightly integrated with the EP matrix. This structural integration not only increases the mechanical interlocking but also improves the chemical bonding at the interface, contributing to the significant enhancement in adhesion strength.

## 3. Discussion

### 3.1. Formation Mechanism of LDH Pretreatment Layer

**Figure 11:**
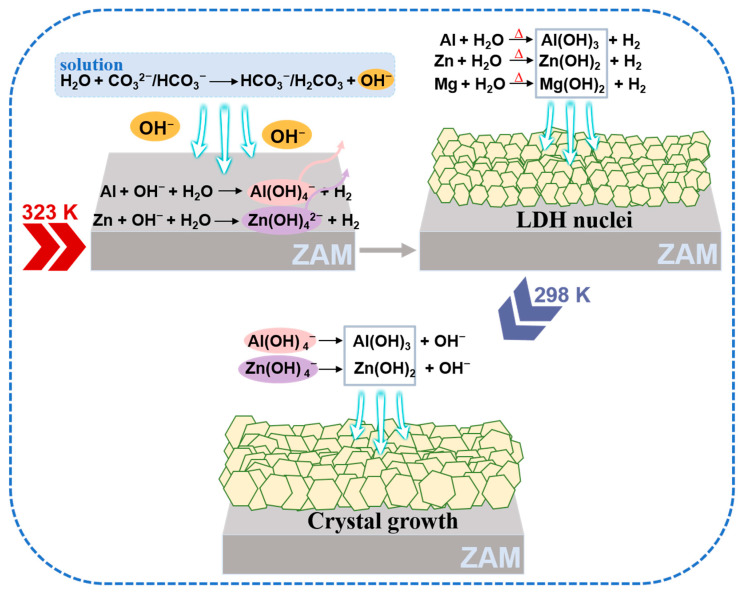
Schematic diagram of the in situ deposition of the ZnAlMg-LDH coating on the surface of ZAM steel without the addition of external film-forming metal salts.

The ZAM coating contains substantial amounts of zinc (Zn) and aluminum (Al), along with trace amounts of magnesium (Mg) [41]. Both Zn and Al are amphoteric metals, which can react with either acids or bases. Leveraging this unique property, a method is devised in this paper that utilizes heat and alkalinity in concert, to dissolve the metals from the coating and deposit them to form LDH conversion coatings. This approach allows for the in situ preparation of ZnAlMg-LDH coatings on the surface of ZAM steel using a hot sodium carbonate solution without the addition of external metal ions. The reaction equations that occurred during the preparation process are summarized in Table 2. Sodium carbonate hydrolyses in water to create an alkaline environment; moreover, the existing CO_3_^2−^ ions can also serve as intercalation ions for LDH formation, aiding in the stabilization of the LDH structure [42]. The deposition process of LDHs, as illustrated in Figure 11, proceeds as follows: initially, CO_3_^2−^ ions in the solution react with H_2_O, generating OH^−^ ions (Equations (2) and (3)), thereby providing an alkaline environment for the dissolution of Al and Zn from the ZAM steel coating (Equations (4) and (5)). Concurrently, under heating conditions, Al, Zn, and Mg react with water, transforming these metals into hydroxide deposits (Equations (6)–(8)), which anchor onto the metal surface and provide nucleation sites for the in situ growth of LDHs. Alternatively, the soluble aluminate and zincate species in the solution transformed into their corresponding hydroxides under cooling conditions (Equations (9) and (10)) promoting the growth of LDH nuclei and resulting in the formation of a uniformly coated LDH pretreatment layer on the metal surface.

The hydrolysis of carbonate ions (Equations (2) and (3)) is crucial for LDH formation, governed by their hydrolysis equilibrium constants *K*_a_ (Equations (11) and (12)). According to the van’t Hoff equation (Equation (13)), *K*_a_ increases with temperature (T), as the reactions (2) and (3) are endothermic (∆H^0^ > 0), elevating OH^−^ concentration. Conversely, lower temperatures reduce pH. Moreover, the OH^−^ concentration further depends on the Na_2_CO_3_ concentration. At a fixed temperature (constant *K_a_*), a higher Na_2_CO_3_ concentration drives Equations (2) and (3) toward the forward direction. As indicated by Equations (11) and (12), this results in an increased OH^−^ concentration and a corresponding rise in pH. Conversely, reducing the Na_2_CO_3_ concentration leads to a decrease in the system’s pH; therefore, when the temperature or concentration of the solution is low, the metals in the ZAM steel coating do not receive sufficient OH^−^ and thermal energy to dissolve, and the LDH lacks the appropriate OH^−^ concentration necessary for its growth. At excessively high temperatures or concentrations, the amphoteric metals (Zn, Al) forming the LDH can undergo dissolution due to the elevated OH^−^ content.

(11) $$K_{a}=\frac{\left[{\mathrm{HCO}}_{3}^{-}\right][{\mathrm{OH}}^{-}]}{\left[{\mathrm{CO}}_{3}^{2-}\right]}$$

(12) $$K_{a}=\frac{\left[H_{2}{\mathrm{CO}}_{3}\right][{\mathrm{OH}}^{-}]}{\left[{\mathrm{HCO}}_{3}^{-}\right]}$$

(13) $$\ln\left(\frac{K_{a}\left(T_{2}\right)}{K_{a}\left(T_{1}\right)}\right)=-\frac{∆H^{0}}{R}\left(\frac{1}{T_{2}}-\frac{1}{T_{1}}\right)$$

### 3.2. Mechanism of Corrosion Protection of the Coating Systems

For LDH conversion layers employed as pretreatment layers, several essential criteria must be met. Primarily, a robust interfacial adhesion between the pretreatment layer and the metal substrate is required. Furthermore, strong bonding between the pretreatment layer and subsequent coatings is necessary. Ultimately, the pretreatment layer must exhibit effective corrosion inhibitor loading capacity and resistance to corrosive ions. Herein, a facile and mild alkaline treatment was employed on ZAM steel, where a short 30 s immersion in a Na_2_CO_3_ solution at merely 50 °C enabled the in situ growth of a vertically aligned LDH pretreatment layer. Figure S3 shows the surface morphology of the LDH and LDH/VOx samples after cross-cut testing and tape peeling. The LDH pretreatment layer remained tightly adhered to the metal substrate. Notably, since no external metal ions were supplied during the process, the ZAM coating itself served as the exclusive source of metallic species. This complete in situ metal ion supply resulted in an unprecedented interfacial bonding strength, thereby establishing an exceptional primary interface for the coating system.

**Figure 12:**
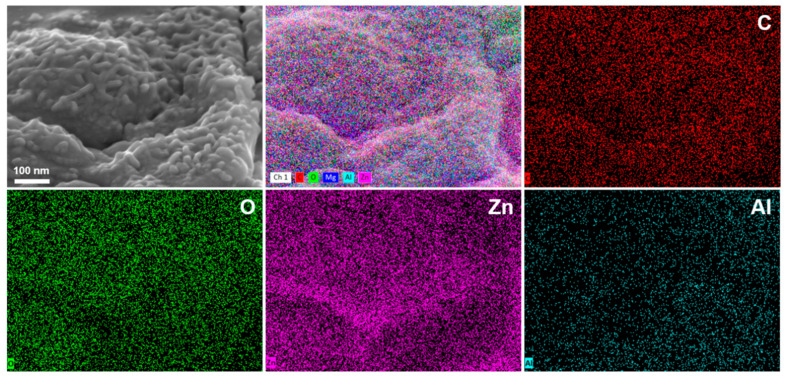
SEM and EDS results of the LDH pretreatment layers after a pull-out test.

The LDH layers are rich in hydroxyl groups [43], which endows them with excellent affinity toward epoxy resin [44]. Furthermore, as demonstrated by Karami et al. [45], the strong interaction between epoxy resin and Zn/Al-LDH hydroxide layers enables Zn atoms to attack the lone pair electrons of oxygen in epoxide rings, participating in ring-opening reactions during resin curing and thereby enhancing the curing performance of epoxy resin. High affinity combined with the ability to promote resin curing results in firm anchoring of the epoxy resin onto the LDH pretreatment layer. In coating technology, the interaction between the resin and the substrate can be categorized into two mechanisms. The first is adhesion, whereby the resin directly adheres to the metal substrate through van der Waals forces, hydrogen bonding, or chemical bonding. The second mechanism involves the presence of an intermediate “adhesive” layer between the resin and the substrate, which forms strong bonds with both materials and thereby enhances the overall adhesion of the coating system. Figure 12 presents the SEM and EDS results of the coating system after resin delamination. On the exposed pretreatment layer surface following outer resin removal, uniformly distributed signals of C and O (from epoxy resin) as well as Zn and Al confirm the tight interlocking between the resin and LDH, forming a “concave-filled structure.” The LDH pretreatment layer serves as an effective adhesive interface, greatly enhancing the bonding between the resin and the substrate and thereby improving its resistance to delamination. The pull-off test results demonstrate a significant increase in adhesion strength (Figure 10). Consequently, a second high-performance interfacial layer is successfully established.

Vanadate ions exhibit excellent corrosion inhibition performance for Al alloys [46,47], which enables their application as high-efficiency inhibitors for ZAM steel. Figure S2 presents the Tafel curves of ZAM steel in a 3.5 wt.% NaCl solution and a 3.5 wt.% NaCl + 0.01 M NaVO_3_ solution, along with corresponding surface morphologies after 24 h immersion. In the NaCl solution, ZAM steel showed a corrosion current density (I_corr_) of 3.67 × 10^−6^ A/cm^2^, while in the NaVO_3_ containing solution, the I_corr_ dramatically decreased by two orders of magnitude to merely 6.81 × 10^−8^ A/cm^2^. A comparison of optical images (c) and (d) after immersion clearly demonstrates that NaVO_3_ significantly enhances the corrosion resistance of ZAM steel in a 3.5 wt.% NaCl solution.

**Figure 13:**
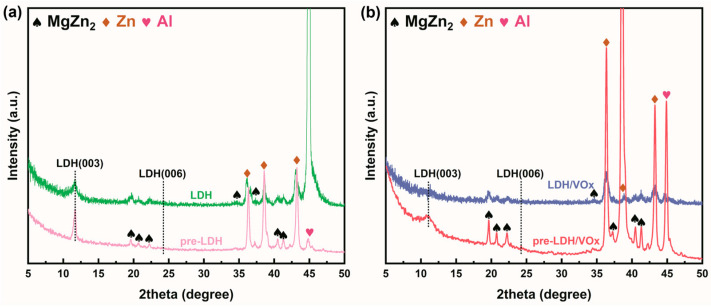
XRD results of (**a**) LDH and (**b**) LDH/VOx samples after 24 h of immersion in a 3.5 wt.% NaCl solution.

In the anion exchange sequence of LDH, the planar structure and strong electronegativity of CO_3_^2−^ prevents conventional Cl^−^-for-CO_3_^2−^ interlayer anion exchange [48,49], which implies that the LDH pretreatment layer prepared in this study cannot capture Cl^−^ during natural corrosion processes. Figure 13a,b present XRD patterns of bare LDH and LDH/VOx samples before and after immersion in a 3.5 wt.% NaCl solution. After 24 h of immersion, the (003) diffraction peaks of both samples show no significant shift, indicating that Cl^−^ did not intercalate into the LDH interlayers and the crystal structure remained unchanged. However, the decreased (003) peak intensity suggests partial LDH dissolution due to exposure to high Cl^−^ concentrations.

### 3.3. Commercial Resin Applications

The LDH pretreatment layer was compared with a commercial phosphating process, over which a commercial epoxy resin (CEP) was subsequently coated. Optical images of the intact commercial EP coating immersed in a 3.5 wt.% NaCl solution are provided in Figure S4. Corrosion spots started to appear on the Blank_CEP sample on the fifteenth day of immersion and continued to expand during further soaking. The P_CEP sample began to show slight corrosion only after the fiftieth day. Both LDH_CEP and LDH/VOx_CEP samples exhibited excellent corrosion resistance throughout the entire 90-day period. This indicates that the LDH pretreatment layer outperforms the commercial phosphating process. The pull-off test results for the commercial resin system (Figure S5) demonstrated that the adhesion strength of the LDH_CEP sample (9.88 MPa) and the LDH/VOx_CEP sample (7.76 MPa) were higher than that of the P_CEP sample (5.78 MPa), while the Blank_CEP sample showed the lowest adhesion strength (1.75 MPa). Therefore, the LDH pretreatment layer can still be effectively utilized in commercial epoxy resin systems, providing them with superior corrosion resistance and coating adhesion.

## 4. Experimental

### 4.1. Materials and Chemicals

The ZAM steels were kindly supplied by Baosteel Co., Ltd. (Shanghai, China) and were cut into a size of 40 mm × 30 mm × 0.5 mm. The ZAM coating consists of 55 wt.% Al, 43 wt.% Zn, 1 wt.% Mg and balance Si. Sodium carbonate (Na_2_CO_3_, AR), sodium chloride (NaCl, AR) and sodium metavanadate (NaVO_3_, AR) were purchased from Aladding Industrial Corporation (Shanghai, China). Isopropyl alcohol ((CH_3_)_2_CHOH, AR) was purchased from Sinopharm Group (Beijing, China). Commercial phosphating solution (DR-378) was purchased from Dingrun Chemical Co., Ltd. (Dongguan, China). Pure epoxy resin (Ep, bis-phenol A, type E-44) and the curing agent polyamide (650) were purchased from Macklin (Shanghai, China). Commercial epoxy resin (9000-B001), curing agent (9000-B001H), and diluter (9000-B001T) were purchased from AA-sund Co., Ltd. (Yangzhou, China).

### 4.2. Synthesis of LDH Conversion Coatings on ZAM Steel

Prior to use, the ZAM steel substrates were ultrasonically cleaned in acetone and ethanol sequentially, each for 5 min, to remove surface contaminants. A series of isopropanol/water (with volume ratio of 3:7) solutions containing sodium carbonate at concentrations of 0.08, 0.12, 0.16, and 0.20 mol/L were prepared. These solutions were subsequently heated to different temperatures (30, 50, and 70 °C). ZnAlMg steel sheets were immersed statically into the pre-heated solutions and allowed to react for 30 s. Then, the obtained samples were alternately rinsed with deionized water and ethanol, followed by drying at 70 °C for 1 h.

### 4.3. Encapsulation of Corrosion Inhibitor (NaVO_3_) on LDH Conversion Coatings

As a commonly used corrosion inhibitor for Al alloys, NaVO_3_ was selected as the corrosion inhibitor for ZAM steel and loaded into the LDH pretreatment layer. A 0.1 mol/L NaVO_3_ solution with pH 8.5 ± 0.1 was adjusted by using a 0.1 mol/L NaOH aqueous solution. The LDH-coated ZAM steel sheets were then immersed in this solution at 25 ± 1 °C for 3 h. The resulting samples were designated as LDH/VOx.

### 4.4. Preparation of Epoxy Coatings

The polymeric coating was prepared using epoxy resin (E44) and a polyamide curing agent. The epoxy resin, curing agent, and solvent were ultrasonically mixed. The mass ratio of epoxy resin to curing agent was 5:4 and the solvent was composed of xylene and n-butanol with a volume ratio of 7:3. The epoxy resin was applied onto bare ZAM steel, LDH-coated ZAM steel, and LDH/VOx-coated ZAM steel using a film applicator (OSP-80/250, Qigong, Shanghai, China). The coated samples were initially cured at 25 °C for 12 h, followed by post-curing at 45 °C for 7 days. The thickness of the epoxy coatings, measured using a coating thickness gauge (Qnix 8500, Cologne, Germany), was maintained at 40 ± 2 μm. The epoxy-coated bare ZAM steel, LDH-coated ZAM steel, and LDH/VOx-coated ZAM steel were designated as EP, LDH_EP, and LDH/VOx_EP, respectively.

### 4.5. Characterization

The X-pert Powder diffractometer (40 kV, 40 mA, CuKα radiation, X’celerator detector, Malvern, UK) was used to examine the phase structure of the LDH conversion coatings. The surface morphology was characterized by scanning electron microscopy (SEM, SU8010, Hitachi, Tokyo, Japan). Laser Confocal Raman spectrometer (LabRAM-HR-evolution, HORIBA Jobin Yvon, Paris, France) was conducted to analyze the composition of the LDH coatings. The elemental composition was characterized by a Field Emission Analytical Transmission Electron Microscope (2100F, Tokyo, Japan). X-ray Photoelectron Spectroscopy (Axis-Supra^+^, Kratos, Manchester, UK) was conducted to determine the chemical compositions of the LDH pretreatment layer.

The adhesion strength of the coatings was measured by the pull-off adhesion test (Defelsko, positest AT-A) according to ISO 4624:2023 [50]. Zn and Al ion dissolution tests after intensive corrosion of artificially scratched coating samples were carried out on an Inductively Coupled Plasma Mass Spectrometer (NexION-2000, Perkin-Elmer, Waltham, MA, USA).

### 4.6. Corrosion Performance Evaluation

The corrosion evaluation of LDH conversion coatings alone and epoxy-coated samples was accelerated using a 3.5 wt.% NaCl solution. Epoxy-coated samples were assessed in both intact (coated) and artificially defective conditions, followed by immersion in the aforementioned NaCl solution. Artificial defects in the coating were introduced according to ISO 17872:2019 [51], with a scratch length of 10 mm and depth reaching the substrate. Optical photographs of the samples were taken at various times throughout the immersion process.

To quantitatively evaluate the corrosion rate of the LDH-filmed samples, potentiodynamic polarization curves were employed. The polarization curves were measured in a 3.5 wt.% NaCl solution using an M273 potentiostat. The potential scan rate and the overpotential were 0.2 mV/s and 100 mV. Electrochemical impedance spectroscopy (EIS) was utilized to monitor the electrochemical corrosion processes of the epoxy-coated samples during the immersion period. EIS tests were conducted in a 3.5 wt.% NaCl solution on a VersaSTAT 3 (Princeton Applied Research, Oak Ridge, TN, USA) with a three-electrode system, where the intact coating samples served as the working electrode, a platinum sheet acted as the counter electrode, and a Ag/AgCl electrode functioned as the reference electrode. The frequency range for the tests was selected from 100 kHz to 10 mHz with a sine wave voltage amplitude of 25 mV.

## 5. Conclusions

A rapid, mild, and environmentally friendly thermal carbonate solution treatment method was employed in this study to prepare the LDH pretreatment layer. The thin film and its formation process exhibit the following characteristics:

- All metal ions required for the formation of the thin film are derived from the substrate itself, eliminating the need for any external addition of film-forming metal ions. This approach significantly reduces preparation costs. The Na_2_CO_3_ solution used not only provides an alkaline environment but also facilitates the formation of LDH with CO_3_^2−^ intercalation. By sourcing all necessary metal ions for LDH formation from the substrate, excellent adhesion between the LDH layer and the substrate is ensured.
- The pretreatment layer obtained via the thermal carbonate solution treatment possesses a distinctive upright-growth morphology of LDH, which greatly enhances the adhesion strength of subsequent coatings.
- The upright growth morphology also imparts a porous structure to the pretreatment layer, ensuring a high loading capacity for corrosion inhibitors.
- The formation process of the LDH conversion coating is remarkably fast; a well-formed coating can be achieved within just a few minutes.

These features collectively contribute to the superior performance of the LDH conversion coating, laying a solid foundation for constructing a highly corrosion-resistant and strongly adherent complete coating system. The unique combination of rapid processing, excellent adhesion, enhanced porosity for inhibitor loading, and upright-growth morphology makes the LDH pretreatment layer particularly suitable for applications requiring high-performance protective coatings.

## Figures and Tables

**Table 1:** The fitting results of potentiodynamic polarization curves.

Sample	E_corr_ (V vs. Ag/AgCl)	I_corr_ (i/A·cm^−2^)
LDH/VOx	−1.075	1.56 × 10^−6^
LDH	−1.162	1.21 × 10^−5^
Blank	−1.168	1.60 × 10^−5^

**Table 2:** Reaction equations involved in the preparation process.

Hydrolysis reaction of carbonate ion	CO_3_^2−^ + H_2_O ↔ HCO_3_^−^ + OH^−^	(2)
	HCO_3_^−^ + H_2_O ↔ H_2_CO_3_ + OH^−^	(3)
Reaction of Zn/Al with alkali	2Al + 2OH^−^ + 6H_2_O → 2Al(OH)_4_^−^ + 3H_2_	(4)
	Zn + 2OH^−^ + 2H_2_O → Zn(OH)_4_^2−^ + H_2_	(5)
Reaction of alloying elements with H_2_O under heating conditions	2Al + 6H_2_O → 2Al(OH)_3_ + 3H_2_	(6)
	Zn + 2H_2_O → Zn(OH)_2_ + H_2_	(7)
	Mg + 2H_2_O → Mg(OH)_2_ + H_2_	(8)
Formation of hydroxides during the cooling process	Al(OH)_4_^−^ → Al(OH)_3_ + OH^−^	(9)
	Zn(OH)_4_^2−^ → Zn(OH)_2_ + 2OH^−^	(10)

## Data Availability

The original contributions presented in this study are included in the article/Supplementary Materials. Further inquiries can be directed to the corresponding author.

## Supplementary Materials

The following supporting information can be downloaded at: https://www.mdpi.com/article/10.3390/molecules30173491/s1, Figure S1: (a) TEM image and (b) EDS point scan results of LDH coating; Figure S2: (a) Tafel plots and (b) fitting results of ZAM steel in 3.5 wt.% NaCl solution (with and without NaVO_3_); (c) Optical images of ZAM steel after immersion in 3.5 wt.% NaCl + 0.01 M NaVO_3_ solution for 24 h; (d) Optical images of ZAM steel after immersion in 3.5 wt.% NaCl solution for 24 h; Figure S3: The surface topography of LDH and LDH/VOx samples following cross-hatch adhesion testing with tape removal; Figure S4: Optical images of the intact commercial coating samples immersed in 3.5 wt.% NaCl solution during 90 days; Figure S5: Pull-off test results of different commercial coating samples.

## Abbreviations

The following abbreviations are used in this manuscript:

ZAM	Zn-Al-Mg
LDH	Layered double hydroxides
