# Supplementary material for: Enhanced Corrosion Performance of Epoxy Coatings Painted on ZnAlMg-LDH Conversion Film Vertically Grown on ZAM Steels from Sodium Carbonate Solution
Source: Molecules. 2025 Aug 25;30(17):3491. doi: 10.3390/molecules30173491 (PMC12430651; doi:10.3390/molecules30173491)
Supplement: Supplementary file 1 [file molecules-30-03491-s001.zip › molecules-3779833-supplementary.pdf]

Supporting information

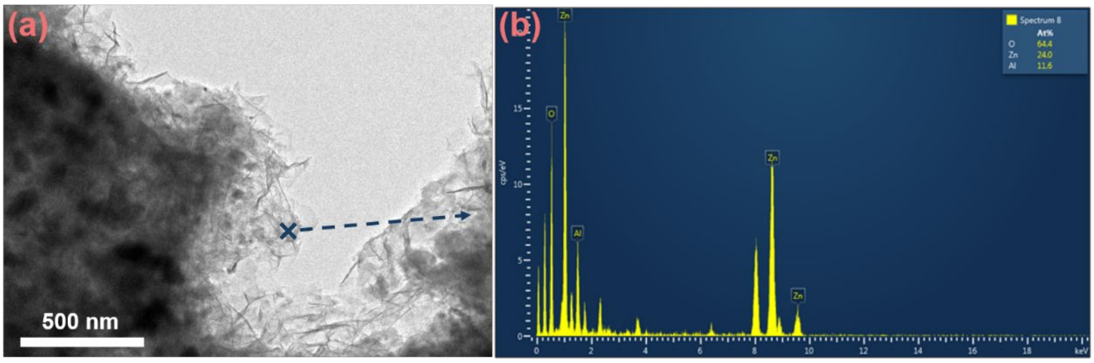

Figure S1. (a) TEM image and (b) EDS point scan results of LDH coating.

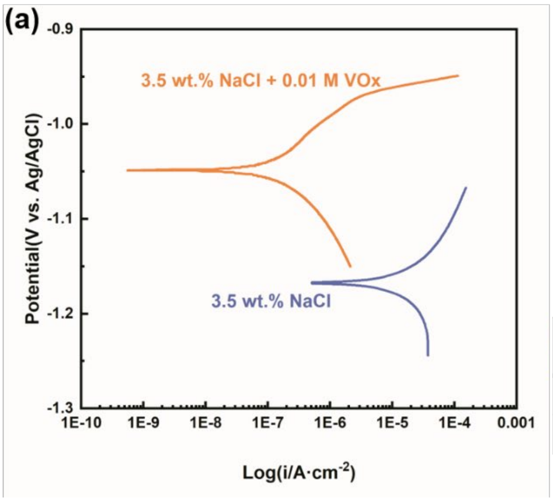

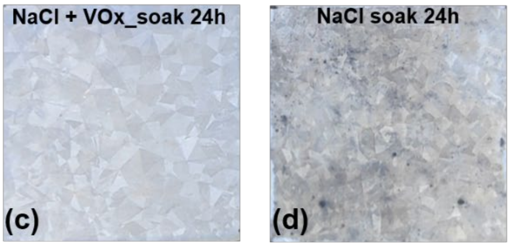

(b)

Sample	E_{corr} (V vs. Ag/AgCl)	I_{corr} (i/A·cm^{-2})
NaCl + VOx	-1.050	6.81E-08
NaCl	-1.168	3.67E-06

Figure S2. (a) Tafel plots and (b) fitting results of ZAM steel in 3.5 wt.% NaCl solution (with and without $NaVO_3$); (c) Optical images of ZAM steel after immersion in 3.5 wt.% NaCl + 0.01 M $NaVO_3$ solution for 24 h; (d) Optical images of ZAM steel after immersion in 3.5 wt.% NaCl solution for 24 h.

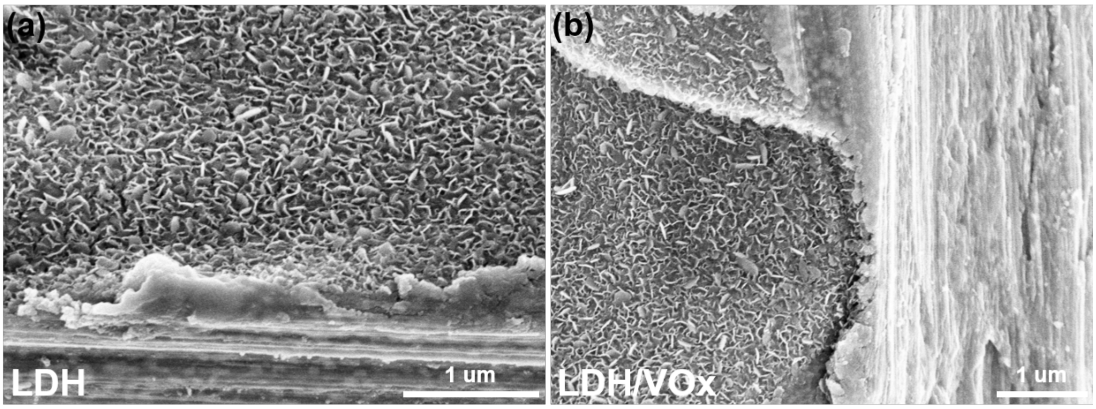

Figure S3. The surface topography of LDH and LDH/VOx samples following cross-hatch adhesion testing with tape removal.

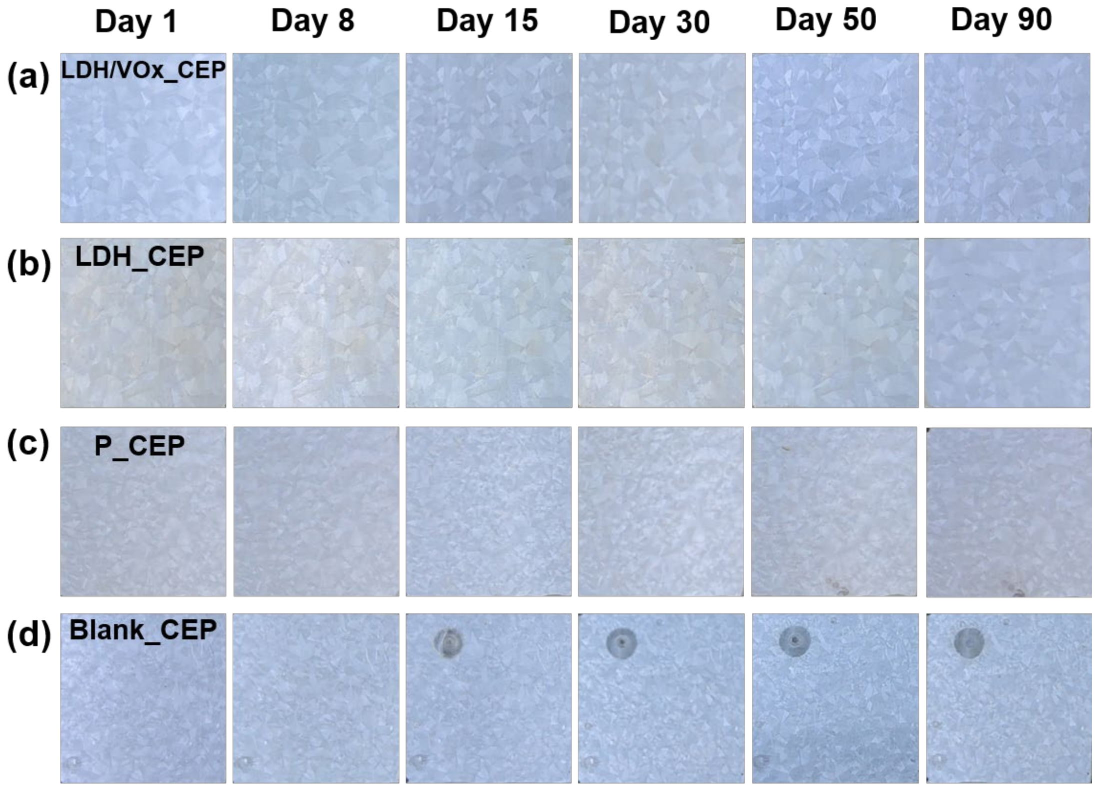

Figure S4. Optical images of the intact commercial coating samples immersed in 3.5 wt.% NaCl solution during 90 days.

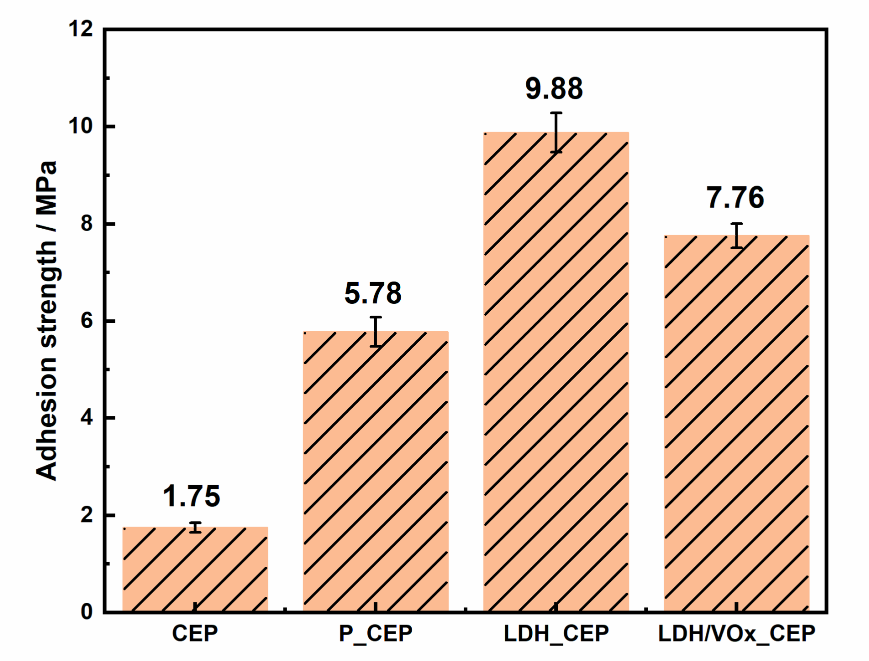

Figure S5. Pull-off test results of different commercial coating samples
